# The knowledge and self-management educational needs of older adults with knee osteoarthritis: A qualitative study

**DOI:** 10.1371/journal.pone.0230318

**Published:** 2020-03-30

**Authors:** Siti Salwana Kamsan, Devinder Kaur Ajit Singh, Maw Pin Tan, Saravana Kumar

**Affiliations:** 1 Physiotherapy Program & Centre for Healthy Ageing & Wellness, Faculty of Health Sciences, Universiti Kebangsaan Malaysia, Kuala Lumpur, Malaysia; 2 Department of Physical Rehabilitation Sciences, Faculty of Allied Health Sciences, International Islamic University Malaysia, Selayang, Pahang, Malaysia; 3 Division of Geriatric Medicine, Department of Medicine, Faculty of Medicine, University of Malaya, Kuala Lumpur, Malaysia; 4 Department of Medical Sciences, Faculty of Healthcare and Medical Sciences, Sunway University, Bandar Sunway, Malaysia; 5 School of Health Sciences, City East Campus, University of South Australia, Adelaide, South Australia; EHESP Paris, FRANCE

## Abstract

Knee osteoarthritis (KOA) is closely related with ageing, physical disability and functional dependency. The course of KOA is considered progressive and irreversible. Engagement with self-management may, however, minimize the impact of KOA. To be fully engaged with self-management activities, knowledge about KOA is a prerequisite. There is limited empirical data on older adults’ understanding on KOA and their information needs about KOA. Therefore, the aims of this study were to explore older adults’ knowledge about KOA and their perspectives on the information required to enable self-management. Three focus groups were conducted with 16 older adults with KOA. The sample consisted of three men and thirteen women with the mean age 73.2 years (range from 61 to 89). Thematic content analysis revealed two themes which were understanding about KOA and information needed about KOA. Participants’ knowledge about KOA varied between individuals with many expressing that they needed more information about KOA. A targeted strategy is needed to educate older adults about KOA in order to support and prepare them for self-management.

## Introduction

Knee osteoarthritis (KOA) has been highlighted as one of the top contributors to global disability [1–3]. Knee pain, joint stiffness and lower limb muscle weakness are the predominant complaints related to KOA which impair mobility, leading to functional limitations [4–9]. The impairments and limitations associated with KOA may lead to psychosocial issues such as anxiety, depression and helplessness [10–12]. As a result, a decline in quality of life is reported by people with KOA [4, 13–15]. The impact of KOA extends beyond the individual level, as it imposes a significant burden on society and health care services [16,17].

The focus of KOA management primarily concerns symptom relief and optimization of functional outcomes [18]. Within management guidelines for OA, self-management education intervention has been recognized as one of the fundamental components for successful management of KOA [19–23]. Such interventions involve acquisition of knowledge and skills in order to empower individuals towards self-management [24]. Providing disease information about KOA is more likely to engage people with KOA in positive behavior change for better health outcomes [25–28]. Even though delivering disease information during initial consultation has become routine, dissatisfaction regarding the details about KOA provided by health providers has been reported in early studies [29–31]. The reported dissatisfaction surrounds vagueness of information, limited consultation time and lack of precision in providing explanations which in turn leads to limited understanding about KOA and poor adherence to self-management [29, 32, 33].

Literature related to the knowledge of and information about KOA among older persons remains scarce [34, 35], especially in a country with multiethnic population such as Malaysia. Given that KOA is a global issue, more studies of diverse population background are needed as these population groups may have different perspectives due to geographical, cultural and lifestyles differences. Addressing this knowledge gap is essential as it will assist healthcare professionals in tailoring self-education management interventions for people with KOA to ensure optimal outcomes. Therefore, the aim of this study was to explore older adults’ knowledge on KOA and their perceptions on the information they need about KOA.

## Methodology

### Study design

This study utilized qualitative research methods in the form of focus group discussions (FGDs). FGD was employed to obtain a variety of information about any particular issues, topics or phenomenon [36]. The conduct and reporting of this research was informed by the consolidated criteria for reporting qualitative research (COREQ) [37] (S1 Checklist).

### Sample population

List of potential participants was obtained from geriatric unit in a selected teaching hospital in Klang Valley in Malaysia. Older adults aged 60 years and above with a clinical diagnosis of KOA and were able to understand English language were included. The diagnosis of KOA was made according the American College of Rheumatology criteria [38, 39] by general physicians, rheumatologists or orthopedics specialists. Those who had other types of arthritis of knee such as rheumatoid arthritis and gout were excluded. Participants were selected through purposive sampling strategy to obtain broad range of information [40], representing all main ethnic groups including Malay, Chinese, and Indian. Selected individuals were first screened through telephone calls and those who fulfilled the criteria were invited to participate. Those who were eligible and agreed to participate in the study were scheduled for focus group discussions.

### Procedure

Participants were invited for the FGDs which were held in a private space at a teaching hospital in the Kang Valley. The FGD sessions were conducted by the same researcher (the lead author), a female physiotherapist with postgraduate qualifications in musculoskeletal physiotherapy and currently enrolled in doctoral studies at a local tertiary educational institution. A field note taker was employed to record the non-verbal behavior and group dynamics. Both researcher and field note taker had no prior relationship with the participants. The questions which underpinned the FGDs were guided by a specific framework [41]. An extensive review of published literature was performed to determine the most relevant topics and questions for the FGD. The draft topics and questions were then reviewed by the research team and consensus development through discussions. These finalized topics were used in FGD sessions (Table 1).

**Table 1:** Interview guide.

Topic	Sample of questions
Definition of KOA	What do you understand by the term KOA?
Pathology of KOA	Can you briefly describe about the process of KOA?
Causes of KOA	What do you think about the causes of KOA?
Consequences of KOA	How much KOA affect people life?
Management of KOA	How do you think people can manage the symptoms of KOA?
Information about KOA	What do you want to know more about KOA?
	We would like to know your opinion about any important information that should be delivered to people who have KOA?
	How information on KOA can help people to manage better their KOA?

KOA, knee osteoarthritis

The FGDs began with a brief explanation about the aims of the study, confidentiality assurance, participants’ right to withdraw from the discussion sessions and their roles in the discussion. Participants were provided with a name card containing their preferred pseudonyms to maintain anonymity. Each session lasted between one to one and a half hours and was audio recorded. FGD sessions were scheduled until the data have reached a point of saturation [42]. To determine when the data has reached saturation point, data collection and data analysis were performed concurrently [43]. A patient information sheet about the study was provided to participants and written informed consent was obtained prior the interviews. Information on the sociodemographic background was obtained prior to FGD sessions.

### Data analysis

Demographic data were descriptively analyzed. The FGD were recorded and transcribed verbatim by researchers and checked by other researchers for accuracy against audio-recordings. FGD transcripts and participants were labeled by unique alphanumeric codes such as T1 for interview transcript 1 and P1 for participant 1. The transcribed data were imported to NVIVO software version 10 as means of data management and were analyzed using thematic content analysis based on Braun and Clark (2006) framework (Table 2). Given the extensive amount of literature in this field, familiarization and initial code extraction were informed by reviewing previous studies on understanding of and information needs about KOA [30, 34, 35]. Inductive approach was then undertaken to identify any new codes, categories and themes. The data was coded by researcher and were cross-checked by other researchers. The field notes which taken by the observer were reviewed as means of cross-checking FGD findings.

**Table 2:** Procedure of data analysis based on Braun and Clark [44] framework.

Steps	Activities
**Familiarization**	Repeated reading of interview transcripts was performed by the first researcher to obtain sufficient understanding of interview statements.
**Initial code extraction**	Each transcript was read and analysed on a line-by-line basis. Statements related with the research questions were identified and coded by one researcher and were cross-checked by the other researchers in the research team.
**Theme search**	Initial codes were grouped into particular categories and collapsed into particular sub-themes. Sub-themes identified were reviewed and examined by the research team.
**Theme review**	Sub-themes containing similar concepts were combined to create themes. Each theme were appraised in the meetings by the members of the research team.
**Theme definition**	All themes were refined and heavily debated among researchers until consensus was achieved.
**Report production**	A report of the findings was prepared for dissemination purposes. Vivid extracts for the themes were presented when illustrating the findings in the report.

The rigor for data analysis was enhanced through member checking and code-recode strategy [45]. For member checking, the initial coded statements were sent to participants through email or messenger application and they were asked to provide their feedback. Reminders were sent to participants if no feedback was received within two weeks. For code-recode strategy, several meetings with the research team were held to review and discuss the coded data. Changes were made if necessary until consensus was reached.

### Reflexivity

Our research team has vast clinical experience in managing older adults with KOA. Client education is a part of the routine management of KOA. Clients’ level of understanding and perceptions about KOA are important information that can improve care provided. In addition, adequate understanding about KOA is beneficial to be able to self-monitor and manage the symptoms among people with KOA. However, Malaysian cultural and social norms and values may influence understanding of and perceptions about KOA and therefore impact how older adults in this country manage their KOA. The FGDs were conducted by a researcher who had no prior relationship with participants, performing the discussion in an informal manner. All researchers were involved in designing the study and interpretation of the data. There was no conflict of interest in the outcome of this study.

### Ethics

Approval to conduct this study was obtained from the institutional review board of the Universiti Kebangsaan Malaysia (reference number: NN-2018-106) and University of Malaya (reference number: 20147–390)

## Results

In total, thirty-five older adults with KOA were contacted and twenty-seven agreed to participate and were scheduled in the FGDs. Three FGDs were conducted among sixteen participants, consisted of three men and thirteen women. The first two FGDs were held among twelve participants with six participants in each group, while the third FGD was attended by four participants. Several scheduled participants did not attend the FGD sessions due to unexpected circumstances which included interim illness, family emergency, unpredicted commitments, transportation issues, working responsibility and other personal reasons. Data saturation was achieved after two FGDs and the third FGD session was conducted to confirm the presence of saturation of the data.

Participants’ demographic characteristics are summarized in Table 3. The mean age of participants was 73.2 years, ranging from 61 to 89 years. Two themes emerged from the analysis were understanding about KOA and information needed on KOA. With member checking, nine participants returned their responses on the coded statements and no additional issues were identified.

**Table 3:** Demographic characteristics of participants.

Characteristics	N	%
Age, mean ± SD (range) = 73.2 ± 7.27 (61–89)		
Gender		
Males	3	18.8
Females	13	81.2
Ethnicity		
Malay	4	25
Chinese	10	62.5
Indian	2	12.5
Marital Status		
Single	1	6.2
Married	10	62.5
Divorce	3	18.8
Widowed	2	12.5
Educational Status		
Secondary	6	37.5
Post-secondary-certificate	4	25
College or university	6	37.5
Duration KOA		
Three to five years	3	18.8
More than five years	13	81.2

SD, standard deviation; KOA, knee osteoarthritis

### Theme 1: Understanding of knee osteoarthritis

This theme answered the question of older adults’ general knowledge on KOA including the definition, development, causes, consequences and its management.

#### Sub-theme 1: Literacy on the nature of knee osteoarthritis

Participants’ knowledge on KOA varied. One participant did not understand the actual meaning of ‘osteoarthritis’ and another two participants informed that they had no knowledge on KOA development. Two participants mentioned that KOA was the result of degeneration of knee structures. Many participants linked the development of KOA with factors such as ageing, occupation, inappropriate footwear, trauma and being overweight.

T3P4: “*What is osteoarthritis in Malay*? *Is it swelling of the joint*?”

T1P3: “*Not much knowledge (about KOA development)*. *Erm*, *not interested in biology…*”

*T2P4*: *“The gel probably (something like) dries up or becomes stiff or just disappears by itself and the bones will start rubbing*, *then it got spur [sic*: *spurs develop]*”

*T2P3*: *“I think those people*, *when they were young*, *at work*, *you walk a lot*. *I was a teacher*, *I was a kindergarten teacher*, *I jump*, *I sit and all*, *then the knee (is) affected*, *I did (some) research*, *some people said that (it’s) your job*”.

Even though most of participants were able to recognize the factors that caused KOA, one participant confessed that she was not aware that factors such as genetics and diet contributed to KOA development.

*T1P2*:” *What about genetically*? *Because it seems to run in the family*, *so I don’t know genetically lead (to KOA)*”.

*T1P2*: *“Don’t know whether diet place a part*, *I don’t know*”

#### Sub-theme 2: Consequences of knee osteoarthritis

Regarding on the knowledge of consequences of KOA, joint pain was mostly stressed by many participants, followed by swelling, joint stiffness, joint instability and muscle weakness. Many participants highlighted that KOA resulted in functional difficulties and psychological distress. Participants described that functional deficits experienced by people with KOA were mainly caused by joint pain and mobility impairment. Psychological distress among people with KOA was related with emotional imbalance and lack of self-acceptance. Few participants also expressed that KOA had significant impact on social participation as people tend to go out less and less likely to be involved in social activities due to knee pain. Some of them claimed that people with KOA also had frustration at having restrictions in mobility and becoming dependent on relatives.

*T2P1*: *“There is of course pain*, *tightness*, *may or may not be able to squat*, *you go down and then you can’t come up or when you’re in certain positions*, *sometimes a bit (of) cramp*, *like that*”.

*T3P2*: *“Sometimes*, *I want to decorate my house*, *clean up the house*, *but I cannot anymore*”.

*T1P6*: *“I have to stop going to many places*, *I just go (to) particular places like temples*, *hospital and home*. *I don’t walk around nowadays because of this pain*, *before this I used to walk at Taman Jaya (a large local park)*”.

*T2P5*: *“…feel very unlucky*. *Why I got this*. *Why this thing attacks me you see*. *Can’t accept it*. *So many years*, *already*. *I had it*, *feel very*, *very*, *very*, *very sad …*”.

Almost fifty percent of the participants expressed their concern about limitation to participate in religious or ceremonial rituals. The activities that people with KOA felt restricted in mostly involve sitting cross-legged on the floor or kneeling. Participants lacked knowledge on adaptation techniques which would allow them to continue in participating in such activities.

*T1P5*: *“I can’t squat and erm especially when I go to the Hindu temple you have to bend your knees and sit (sit cross-legged)*”.

*T1P4*: *“If anybody wants to invite me to the house (attending traditional ceremonies)*, *to sit on the floor*, *I said very sorry I cannot*, *once I sit on the floor*, *if I have to get up I need help*, *not only left hand*, *you help on right hand you know…*”.

#### Sub-theme 3: Symptoms management

With regards to the knowledge on symptoms management, participants were able to identify diverse treatment strategies for the management of KOA symptoms. They had described multiple treatments which were suggested either by physicians, family members or their peers. For pharmacological intervention, despite being prescribed appropriate pain medication or supplements by physicians, some doubt towards pharmacological therapy and refused and doubt to take prescribed medications due to their personal perception on the long term effects of medication or experience with the side-effects of medication.

*T3P1*: *“Erm*, *they (doctors) gave me Celebrex (a non-steroidal anti-inflammatory drug)*, *I can’t take Celebrex*, *I had vomiting and giddiness and all*, *too strong…*”.

According to participants, alternatives therapies, including traditional remedies such as salt, herbs, and traditional oils were considered as beneficial approach for people with KOA and hence trialed by many. Family or peer recommendation and personal beliefs were the main reasons for adopting this approach.

*T1P6*: *“Someone tell me you know*, *put salt (on the knee)*, *“you better put salt*, *will be good”*, *I try that all the time*”.

*T3P2*: *“You take the ginger*, *about one a grasp (fistful)*, *you blend it*, *then you put it on the knee and then wrap it (the knee)*. *Before sleep*, *you do*, *next morning you will feel better*. *I used to do that if I my feel my knee very bad*. *Three nights I did*, *and then I feel a bit better*”.

Physiotherapy interventions were other conservative approaches identified by participants. Most had received physiotherapy treatment and agreed that physiotherapy interventions were important in reducing the impact of KOA. Nevertheless, those who had experience with rehabilitation also expressed that the temporary effects of treatment. Many also complained of feeling exhausted after treatment sessions, and being tired of having multiple treatment sessions.

*T2P5*: *“Yes*, *the physio is maintained*, *if you do your physio*, *it will maintain (symptom control)*, *it won’t become worse*, *it will be maintaining like what you are having now you see…*”.

*T3P1*: *“Erm physiotherapy is good*. *But after sometime*, *(the symptoms) come back again*”.

Several participants suggested that instability of the knee due to KOA could be managed with knee supports and walking aids. They claimed that both knee supports and walking aids improved their confidence and comfort when performing daily chores. Conversely, one participants had a different perspective on knee guard, and expressed his concern on the long term effects of or over reliance on knee guard.

*T1P4*: *“I am wearing the knee guard on the knee*. *I feel safe when I go up the bus and climb up the hill*, *it sorts of gives you confidence*”.

*T2P4*: *“I walk with aids you know*, *I walk with the third leg*, *help me a lot*. *I found that aids help me*”.

*T1P5*: *“People will become too dependent on it (knee guard)*, *without that you won’t be able to walk*”

Many participants were aware of or had been offered intraarticular treatments by their physicians. However, several participants expressed doubts regarding the perceived benefits from such injections. Almost all participants however refused to receive any invasive management because they had fear of surgery. Only two participants mentioned the necessity of total knee replacement (TKR) as they were aware that KOA was irreversible and hence believed that TKR was the only solution.

*T3P3*: *“I went to see the doctors when I had the pain*, *(doctor suggest) operation*, *injection*, *but I don’t want*, *injection I don’t want*, *operation I also don’t want*”.

T2P5: *“I don’t think so (recovery)*. *The operation only you can heal*. *Only take operation other than that not much you can do for it*”

Despite of having challenges in performing spiritual activities, several participants highlighted the roles of spiritual activities in modifying the effects of KOA. Several participants believed that faith through their religious practices gave them hope and solace to endure and accept their condition. One participant believed that movement during their routine prayers were beneficial for the flexibility of the knee joint.

*T3P2*: *“*…*we read verses in Quran for knee pain*, *bone pain*, *everything*, *we try*, *just trust in God*, *read the verse a lot*, *like Ustazah (a female teacher) said*, *do exercises*, *pray a lot*, *at the end*, *it disappears*”

*T2P3*: *“I do research and check*, *then I see the praying activities do a lot because they need to bow and to kneel*, *it is good joint exercise*, *you know*.”

### Theme 2: Information needed on knee osteoarthritis

This theme explored the information needs related to KOA. Disease information, self-management skills and guidance on healthy lifestyle were identified as the information needed among older adults with KOA.

#### Sub-theme 1: Disease information

Participants had strong desire to learn more about KOA. Even though they had been living with KOA many years, some would like to be informed about the nature of KOA including the causes of KOA. They also expressed a need to better understand KOA management though they have had experienced of various forms of interventions.

*T3P2*: *“(I) Want to know more about this problem (KOA)*”

*T1P4*: *“Ya*, *I want to know what the causes of osteoarthritis (are)*.”

*T2P3*: *“I want to know about how to go about without operation*”

*T1P5*: *“All I want to know is how to manage it*, *how to heal it if possible*, *how you are going to sort of manage the pain*, *make sure it doesn’t get worse*, *that’s it*”

#### Sub-theme 2: Self-management skills

Majority of participants would like to learn about possible pain management options without pharmacological intervention. They preferred not to rely on medication because they were anxious about the long term effects of medication and were believed that the pain could be managed by other alternatives. They were also interested to develop self-management skills which might help them to manage their symptoms, to make decisions and to have control of their KOA.

*T1P3*: *“If you have it (KOA) how to manage it*. *What are the things you can do*, *(and) you cannot do*”

*T1P2*: *“What we can we do to keep it under control…*”

*T2P4*: *“Again I want to know how to manage the pain without taking pain killers*. *The pain killers lead to gastric (pain) and you know all kinds of other things*. *That’s all*. *Self-practice*, *that one*, *self-practice to control*”

#### Sub-theme 3: Guidance on healthy lifestyle for KOA

Participants expressed the need for guidance in relation to weight management and exercise. Many participants repeatedly enquired about appropriate types of exercises which are effective yet convenient to perform. Some of them confessed that they forgot most of the exercises prescribed by the physiotherapist because they didn’t do the exercises consistently. A few participants were aware of the importance of practicing a healthy lifestyle, but they did not practice it due to lack of knowledge.

*T1P2*: *“What are the exercises we should do and when*, *the things you know*, *certain movement(s) you shouldn’t do*.”

*T2P4*: *“I think*, *will [sic*: *should] teach them [sic*: *people with KOA] some exercises which they can do at home without special equipment*, *I mean using chair or table to hold*, *exercise (which) doesn’t require special equipment if possible then you do at home*”.

## Discussion

This study was undertaken to address an important knowledge gap in the literature and to provide an insight into older adults’ knowledge and their need for information on KOA. This study found that even though participants in this study have been living with KOA for many years, their lived experience with KOA may not be sufficient to help them to understand and to manage their condition. Interestingly, some of participants were influenced by family, peers and personal beliefs to manage the KOA.

Older adults in this study associated KOA with ageing, occupational demands, inappropriate footwear, previous injury and being overweight. Similar results have been found in previous studies [46–48], suggesting that some individuals with KOA are aware that KOA is related to wear and tear and also abnormal loading of the knee joint. Pain, swelling, stiffness and tightness accurately identified as the main manifestations of KOA in our study. These symptoms have been consistently reported among adults with KOA in previous studies [46, 49].

As cartilage tissues are depleted in KOA, any position with deep knee flexion, which is required for kneeling, would increase the contact pressure on exposed bony surfaces which can lead to joint pain [50]. The effects of KOA on physical function were a primary concern of our participants above pain symptoms, for which many were reluctant to accept oral analgesics. Other qualitative studies had previously reported the negative impact of KOA symptoms on activities of daily living [34, 47, 51, 52]. In addition to activities of daily living, the ability to participate in social and cultural activities concerned our study participants. From the Asian cultural perspective, kneeling and sitting cross-legged are customary practices in most of their traditional and social activities [53, 54]. Participants who are unable to perform these movements voluntarily avoid these settings in order not to stand out or due to fear of being judge by or being a burden to others. Limitation of social and cultural participation may have a psychological impact thereby resulting in psychological distress [47]. Adaptation strategies have been suggested as a solution which protects the knee joint while still being able to continue participating in these activities [5, 55]. Older adults with KOA in our study were, however, not able to provide appropriate coping strategies in order to ensure their continued participation in social and cultural activities. This highlights a gap in clinical practice which takes into account these unique socio-cultural needs of these communities.

Conservative management strategies not involving intra-articular injections or surgical interventions were preferred. Some were reluctant to accept prescription drugs, often resorting to traditional home remedies due to skepticism on the long-term effects of the`medication. The ambivalence towards the pharmacological approach is common among individuals with KOA, particularly within the older population [29, 48, 52]. Alternative medicine is popular among Asian older adults (56) as it is believed to be harmless [56, 57]. The effectiveness of traditional remedies for knee pain, however, remains controversial [58–62]. The reluctance to accept painkillers appeared to lead to activity avoidance and functional limitations, which are the primary concerns of our older persons with KOA as well as being worried about not being able to observe religious rituals involving kneeing or sitting cross-legged. Furthermore, while many of the older individuals who participated in the FGD acknowledged the benefits of exercise and weight reduction, they were unsure how to practice the healthy lifestyle. Recent study identified that pain, physical limitation and lack of motivation were the main barrier for exercises while high-calories eating habit was the main barrier for healthy eating [63].

The effects of chronic disease on the ability to participate in religious activities have previously been reported [64, 65]. Older adults with KOA persisted with religious rituals despite the difficulties encountered. One of the participants believed that repetitive movements involving knee bending while praying was in fact beneficial in maintaining knee range of movement, flexibility and preventing worsening of KOA symptoms. Repetitive kneeling during prayer improves elasticity of the soft tissues, promote activation of the quadriceps muscle and restores knee mobility [66]. However, worshippers who unable to sit on the floor while performing full knee flexion activities can practice an adaptation approach to prevent aggravation of KOA symptoms [5, 55]. Some of our participants did not appear to be practicing adaptation in their ritual activities, hence excluding themselves from this activity. While few others participants mentioned the utilization of chair while performing the prayers. Religious practices also provided spiritual support to our participants and form part of their coping mechanism [67, 68]. Coping strategies which include spiritual support also reduced the emotional impact of KOA [69].

Participants in our study had demonstrated knowledge on the clinical presentation of KOA having lived with the condition for more many years. Previous reports have reported a desire among individuals living with KOA for more knowledge particularly about the management of related symptoms [30, 70]. Providing sufficient information about KOA to clients are particularly vital in supporting decision-making, promoting positive behavioral changes and improving health outcomes [71–73]. However, the knowledge-based of our participants were appeared to be limited to the disease process and symptoms rather than management strategies including symptom control and prevention of disease progression.

The desire to obtain skills in KOA symptom self-management, especially pain was consistently expressed in previous studies [74, 75]. Self-management education (SME) helps with mitigating KOA symptoms and nurturing positive behavior [27, 28, 76, 77]. The findings of this study could inform the contents of future SME intervention programs, while acknowledging the limited transferability as we recruited participants from only one urban city in Malaysia.

### Limitations

As with any research, this research too has some limitations. Firstly, this study was conducted in only one urban city where the majority of the residents are English speaking and of Chinese ethnic. Higher proportions of ethnic Chinese live in the urban cities in Malaysia, which is partly as a result of Malaysia’s colonial history. Different range of views and perspectives about KOA and the information needs, if any, may have been obtained from suburban and rural population, where other ethnic communities are represented. Secondly, more female participants were involved in our FGDs. This can be expected because KOA in Malaysia is reported to be more prevalent in females compared to males [78]. Finally, although FGDs may create a good discussion about a particular topic, it could be a barrier to some participants to highlight certain issues due to fear of disagreement. As means of addressing this, prior to the session, ground rules for FGD were agreed to, participants were asked to respect each other’s views and opinions and were encouraged to be open about sharing their views and opinions.

## Conclusion

Older adults with KOA showed good understanding of disease pathogenesis and clinical manifestations of disease. The acceptability of pharmacological agents, intra-articular injections and joint replacement was limited, influenced by a range of factors. On the other hand, many would resort to traditional home remedies. The fear of functional dependence and inability to perform cultural and religious rituals which require kneeling or sitting cross-legged on the floor were the primary concern. Information about disease process, self-management skills and guidance on healthy lifestyle was highlighted as key topics to be included in a potential SME program, which could be incorporated in future interventional studies so that targeted strategies which meet the needs of the individuals are implemented in clinical practice.

## Supporting information

S1 Checklist Consolidated criteria for reporting qualitative research (COREQ) checklist. (PDF)
